# Multicontrast 3D automated segmentation of cardiovascular images

**DOI:** 10.1186/1532-429X-18-S1-O114

**Published:** 2016-01-27

**Authors:** Matthew Bramlet, Anthony G Christodoulou, Brad Sutton

**Affiliations:** 1 Pediatric Cardiology, Children's Hospital of Illinois, University of Illinois College of Medicine at Peoria, Peoria, IL USA; 2 Bioengineering, University of Illinois College of Engineering, Urbana, IL USA; 3 Beckman Institute for Advanced Science and Technology, University of Illinois, Urbana, IL USA

## Background

Our work explores the use of multiple contrasts to perform automated 3D cardiovascular image segmentation, with the goal of facilitating 3D assessment of subject-specific heart models.

The signal intensity of MR images is a function of intrinsic tissue parameters (e.g., spin density *ρ*, *T*_1_, and *T*_2_), and chosen sequence parameters (e.g., flip angle, *T*_R_, *T*_I_). MR images do not directly measure the underlying states of the tissue parameters, but rather provide observations of functions of the underlying states. When sequence parameters are selected properly, the contrast between tissue types is high enough to allow differentation of tissues via image segmentation.

Our work explores image segmentation using multiple image contrasts (i.e., multiple acquisitions using different sequence parameters). With multiple contrasts, the signal intensities from different images can be used to form a multidimensional feature space, increasing the distance between observations of different tissues as compared to a one-dimensional feature space based on a single image contrast.

## Methods

**Figure 1:**
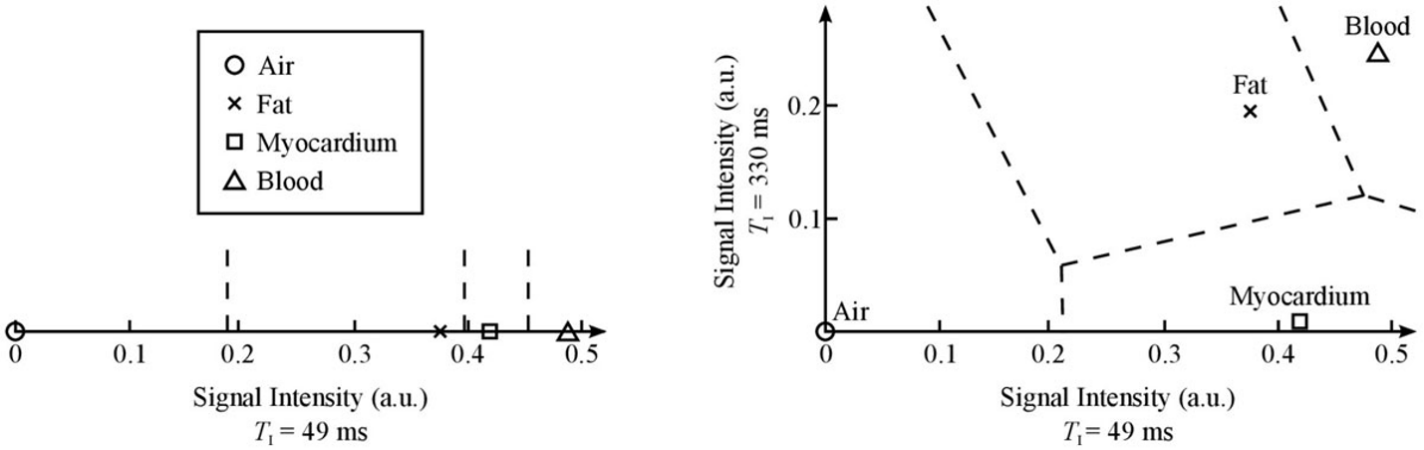
***Left*** **, signal intensities of air, fat, myocardium, and blood using one contrast only**. *Right*, signal intensities of the same tissues in a multidimensional feature space using both contrasts.

We simulated the signal intensity of each tissue for an IR-bSSFP pulse sequence at 1.5 T using common tissue parameters where air was assumed to have zero signal in addition to the following sequence parameters: flip angle = 65°, *T*_R_ = 4 ms, and a range of inversion times from 48 ms to 540 ms. Figure 1 illustrates the advantage of using multiple contrasts for separating tissues, depicting the signal intensities and example separation boundaries between tissues for: *left*, a single contrast, using *T*_I_ = 49 ms; and *right*, two contrasts, using *T*_I_ = 49 ms and *T*_I_ = 330 ms.

## Results

A proof of concept 3D IR-bSSFP scan was collected with different inversion times; all other parameters were the same for both scans using *T*_I_ = 49 ms and *T*_I_ = 330 ms. Images were processed using the FMRIB Software Library (FSL). Images were registered with the FLIRT tool, employing mutual information as the similarity metric, then cropped to include only the heart. The cropped, registered images were segmented by inputting both contrasts into the FAST tool. The number of tissues was specified as four, with the intent of differentiating air, fat, blood, and myocardium.

**Figure 2:**
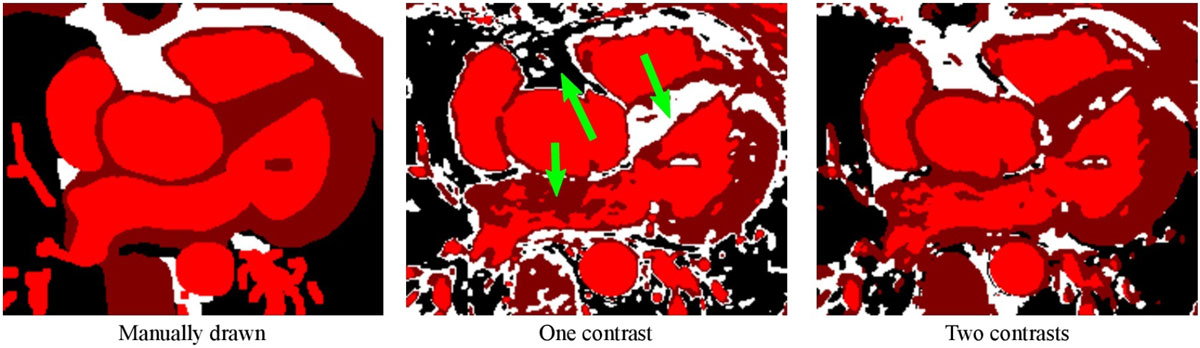
***Left*** **, manual segmentation of tissues (for reference)**. *Center*, FAST with one image contrast. *Right*, FAST with two image contrasts. Arrows indicate large areas of error when using only one image contrast.

Figure 2 shows segmentation results using: *left*, manual drawing (for reference); *center*, FAST with one image contrast (*T*_I_ = 49 ms); and *right*, FAST with both image contrasts (*T*_I_ = 49 ms and *T*_I_ = 330 ms). Using only one contrast results in large areas of tissue misclassification, as indicated by the arrows. The use of multiple image contrasts results in vastly improved tissue classification.

## Conclusions

As more advanced 3D viewing tools become available, the need to automate the segmentation process grows. This simple technique demonstrates a shift in imaging goal from high resolution 2D acquisition to tissue identification and lays the foundation for future sequence design.

